# Skin Necrosis Distal to a Rapid Infusion Catheter: Understanding Possible Complications of Large-bore Vascular Access Devices

**DOI:** 10.7759/cureus.3854

**Published:** 2019-01-08

**Authors:** Wesley H Chou, Tanya N Rinderknecht, Paul K Mohabir, Andrew W Phillips

**Affiliations:** 1 Miscellaneous, Harvard Medical School, Boston, USA; 2 Surgery, Stanford Hospital and Clinics, Stanford, USA; 3 Internal Medicine, Stanford Hospital and Clinics, Stanford, USA; 4 Emergency Medicine, University of North Carolina, Chapel Hill, USA

**Keywords:** rapid infusion catheter, ric, complication

## Abstract

Rapid infusion catheters (RICs) allow expedient conversion of peripheral intravenous (PIV) catheters to peripheral sheaths; however, little is known about potential complications. In this case, a 64-year-old male polytrauma patient had a 20-gauge PIV catheter in the right cephalic vein upsized to an 8.5 French (Fr) RIC without incident during an arrest with pulseless electrical activity (PEA). On RIC post-placement day two, the patient developed edema and ecchymosis extending from the right dorsal mid-hand to the antecubital fossa, just distal to the RIC insertion point. Compartments were soft; the volar surface (including an arterial line location), fingers, and upper arm were normal. The RIC flushed and returned blood appropriately. Ultrasound revealed a noncompressible cephalic vein either related to the catheter or thrombosis, and imaging of the hand showed an ulnar styloid fracture and a minimally displaced triquetral fracture. The RIC was removed immediately. Over the next week, the areas of ecchymosis developed bullae and then sloughed, leaving open wounds extending into the dermis. The patient later expired from unrelated causes.

The area and timing of the skin necrosis were highly suspicious for a catheter-associated complication, despite the presence of the arterial line and small distal fractures. The necrosis was potentially due to thrombosis of the superficial venous outflow system, leading to congestion and skin compromise, but we found no similar reports. Alternatively, the catheter may have ruptured the vein and caused a gravity-dependent ecchymosis, but the volar surface was not impacted, and the catheter was functioning properly. The RIC may also have encroached on the arterial space, decreasing flow, but we would have expected distal hand changes. The only published reports we could find on RIC complications involved a lost guide wire, fragmentation of a catheter during placement, and a case of compartment syndrome, raising the question of whether skin necrosis is truly a rare event or simply underreported with the RIC. Although the exact causal relationship remains unknown in our case, RICs should be removed as soon as possible after immediate stabilization.

## Introduction

Rapid infusion catheters (RICs) allow for large-bore peripheral access in emergent situations requiring timely large-volume infusions. They are short, large-caliber (7 or 8.5 French (Fr)), peripheral catheters placed by upsizing existing traditional peripheral intravenous (PIV) catheters using a Seldinger technique (unpublished study on file at Arrow International, Incorporated: Cavallaro DL, Rosemurgy AS, Michlin JP, Bronleewe SH). RIC use is reported with great success in a broad variety of clinical settings and populations: trauma (adult and pediatric; civilian and military), code resuscitations, obstetric bleeding, and liver transplant cases are the most commonly cited [1-5]. RICs are appealing for their ease and speed of placement (conversion from an existing PIV catheter), their large caliber allowing for up to 750 mL/min infusion rates (unpublished study on file at Arrow International, Incorporated: Cavallaro DL), and their lack of central line-associated complications. The most common complications in antecubital PIV catheters include thrombophlebitis, infection, and hematoma [6]. Despite the extensive innervation of the arm, peripheral nerve injuries from insertion of PIV catheters are rare [7]. Infusion of fluids under excessive pressure can also result in infiltration, which can lead to compartment syndrome in severe cases [8]. However, to date, little is reported in the literature about the risk of complications in RICs. We report a case of skin necrosis of the dorsal forearm distal to a right antecubital RIC.

## Case presentation

A 64-year-old male patient was admitted after a fall from four stories with multiple orthopedic injuries in the chest, back, pelvis, and extremities. On post-injury day one, he underwent pelvic fixation complicated by an arrest with pulseless electrical activity (PEA). During the code, his right antecubital fossa 20-gauge PIV catheter was upsized to an 8.5 Fr RIC without immediate complication. This patient also received placement of a right radial arterial line. The patient received vasopressin and epinephrine after his PEA arrest, but was weaned off of these medications within 24 hours.

**Figure 1:**
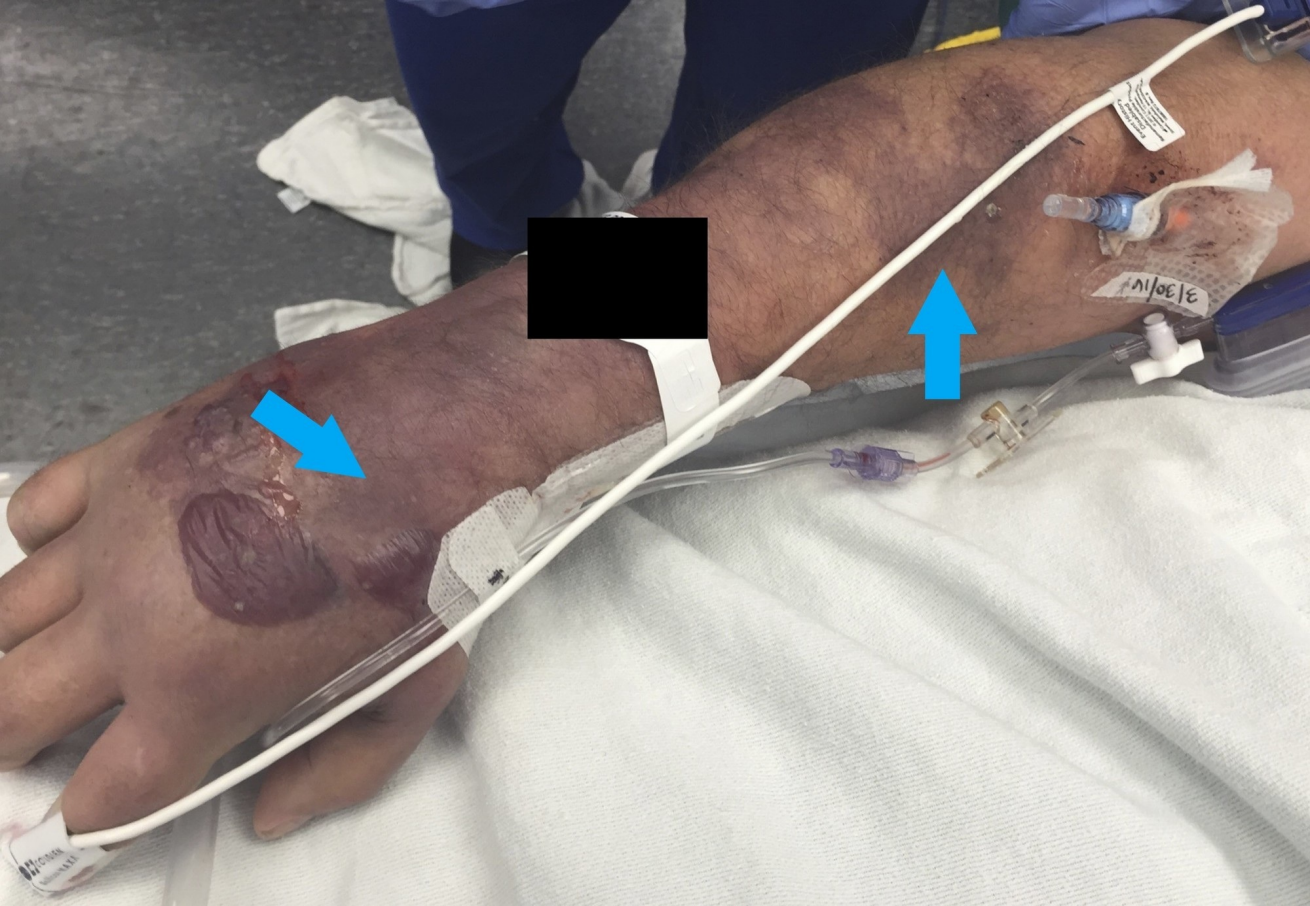
Post-injury day two findings Sites of ecchymosis (blue arrows) just distal to the catheter site but not involving an arterial distribution were present and extended down the patient’s dorsal hand and forearm.

On RIC post-placement day two (post-injury day three), the patient’s dorsal hand and forearm became swollen with ecchymosis extending from the hand to the level of the elbow, while the upper arm and volar surfaces remained normal (Figure 1). The patient's compartments were soft and the neurovascular exam was normal. All skin findings were isolated to the right arm.

Ultrasound revealed a noncompressible cephalic vein, related to either the catheter or thrombosis, and imaging of the hand showed an ulnar styloid fracture and a minimally displaced triquetral fracture.

**Figure 2:**
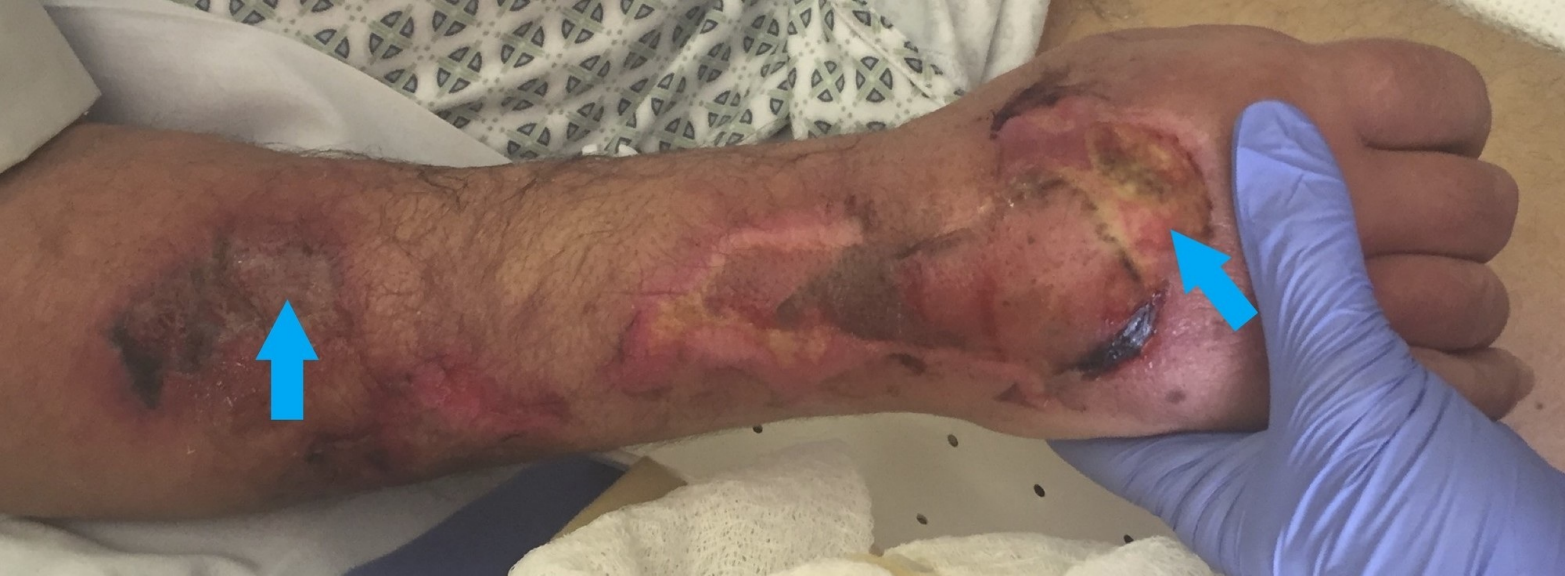
Post-injury day seven findings Areas of skin necrosis (blue arrows) extending to epidermis and dermis were present and slow to heal after debridement.

The RIC was eventually removed, even in the setting of appropriate flushing and blood withdrawal. Over the next week, the areas of ecchymosis progressed to bullae and sloughing. Skin necrosis extended to the epidermis and dermis (partial thickness) of two-thirds of the dorsal forearm and hand, requiring debridement and local wound care (Figure 2). This patient’s forearm wounds continued to heal but he ultimately expired from other causes.

## Discussion

The location and timing of the skin necrosis in our case is highly suspicious for a catheter-associated complication. We considered several possibilities and posit that the most likely explanation is thrombosis of the superficial venous outflow system, leading to congestion and skin compromise. Ligation of veins at this level is usually well-tolerated and performed frequently in vascular surgery, but the distribution, in this case, seems to implicate venous compromise. Alternatively, the catheter may have ruptured the vein and caused a gravity-dependent ecchymosis, but the gravity-dependent volar surface was not impacted and the catheter was functioning properly. The RIC may also have encroached on the arterial space and decreased flow, but we would have expected distal hand changes. Additionally, the patient received the vasoactive medications vasopressin and epinephrine after his PEA arrest, but these were unlikely to have contributed to the skin findings. The skin changes were relatively proximal, and these medications were weaned quickly prior to skin changes. The lack of distal hand changes also speaks against a complication with the previously placed radial arterial line on the ipsilateral wrist. The bony injuries were too small and distal to explain the more proximal changes, and the timing of skin complications was not consistent with immediate traumatic insults such as burn or friction injury.

The RIC in our case was used according to manufacturer instructions in a vein large enough to accept the large-bore catheter. The RIC remained in place for only two days and notably, there is no manufacturer’s recommended time limit for the catheter [9].

There is sparse literature specific to RICs to inform safest use. The Food and Drug Administration's (FDA) Manufacturer and User Facility Device Experience (MAUDE) database describes two case reports; one involved retention of a guidewire in a patient’s arm, while the other involved fragmentation of a catheter during placement with embolization of pieces to pulmonary circulation [10-11]. Another report describes a case of compartment syndrome of the arm after pressurized infusion through a RIC, with the hypothesis that the untapered large diameter catheter may predispose veins to rupture during cannulation or under high infusion pressure [12].

## Conclusions

RICs allow for simple and timely peripheral vascular access with subsequent fast, large-volume fluid infusions to critically ill patients in a range of settings. The clinical course of localized skin necrosis in our patient’s case is concerning for a serious RIC complication, given that no fracture or other device was temporally or spatially related to the site of necrosis. Limited reports of RIC complications in the literature leave the question of whether complications are truly rare or perhaps simply underreported. However, the clinical course of our patient reinforces that RICs should be removed as soon as the patient is stabilized and more definitive access can be established. Further studies are also warranted to establish a safe time frame for the catheter to remain in place.
